# The Development and Psychometric Properties of the Visuospatial Working Memory Assessment (VWMA) for Children

**DOI:** 10.1155/2020/8736308

**Published:** 2020-03-17

**Authors:** Thitiya Wangkawan, Cynthia Lai, Peeraya Munkhetvit, Trevor Yung, Supaporn Chinchai

**Affiliations:** ^1^Department of Occupational Therapy, Chiang Mai University, Thailand; ^2^Department of Rehabilitation Sciences, The Hong Kong Polytechnic University, Hong Kong; ^3^Applied Cognitive Neuroscience Laboratory, Department of Rehabilitation Sciences, The Hong Kong Polytechnic University, Hong Kong; ^4^University Research Facility in Behavioral and Systems Neuroscience, The Hong Kong Polytechnic University, Hong Kong

## Abstract

The visuospatial working memory plays a crucial role in the occupational performance of children including daily living and academic achievement. Unfortunately, relevant visuospatial working memory tests in the occupational therapy setting are lacking. Therefore, it is of clinical interest to develop new assessment tools in this area. The present study is aimed at summarizing the development of the visuospatial working memory assessment (VWMA) and assessing its psychometric properties. The results revealed that the score of item-objective congruence index (IOC) was 1.0 in overall items of assessment. The Cronbach alpha test confirmed that the internal consistency of VWMA showed good reliability in both types of the assessment, with the total score of computerized tests being .88 and the tabletop tests being .81. The computerized test was found to have excellent test-retest reliability with intraclass correlation coefficient (ICC) values ranging from .88 to .99. The tabletop test was found to have a fair to good test-retest reliability with the ICC values ranging from .51 to .63. As regards construct validity, the results revealed that the tasks in the computerized test identified a significant difference between the control group, normal children, and children with attention deficit/hyperactivity disorder (ADHD) group. The exception to this was the *N*-back task in which the independent sample *t*-test of computerized test ranged from 1.61 to 6.23. The results of the tabletop test revealed a significant difference between normal children and the children in the ADHD group over all tasks in which the independent sample *t*-test ranged from 3.05 to 8.40. In conclusion, good psychometric properties established as regards content validity, internal consistency, test-retest reliability, and construct validity provide evidence to support the position that the new VWMA is appropriate for children.

## 1. Introduction

Working memory can be described as a system of the brain, which momentarily stores and manipulates the information that is needed for the complex cognitive tasks of comprehending language, learning, and reasoning [1]. The mechanisms and processes which hold the mental representations of most current urgency for ongoing cognitive tasks available for processing are also part of working memory [2]. A reasonable inclusive definition is therefore the comparatively small body of information that a person can retain in mind, attend to, or, in technical terms, maintain in a state that can be quickly accessed at a single time [3]. Working memory is pivotal to the complex activities and occupational performance of children. At this crucial stage of development, working memory capacity is significantly associated with processing speed, attention span, language, writing, and mathematics [4–6].

In particular, visuospatial working memory is the main part of working memory responsible for storage of visual information. It allows people to begin a journey and turn at the correct point to arrive at their destination safely. This is particularly the case when in an unfamiliar context [7–9]. Visuospatial working memory can be classified into a spatial component (e.g., memory of where the landmark is situated) and a separate visual component (memory of objects and their visual characteristics, such as color and surfaces) [10, 11]. Research has shown that visuospatial working memory is strongly linked to academic achievement in children, especially as regards early visuospatial working memory development of preschool-aged children [12]. According to a previous study by Ricle and colleagues [13], processing speed was found to have a direct impact on the performance of visuospatial working memory tasks, mediated by visuospatial working memory. This implies that the storage and concurrent processing capacity of visuospatial working memory partially mediates the relationship between the facility of a response to a visuospatial stimulus and ultimately to general intelligence.

Children with visuospatial working memory deficit exhibit poor working memory profiles including those associated with mathematics and arithmetic problem solving, [14, 15]. Mathematical achievement is an especially significant factor in the prediction of future academic performance, career progression, and occupational outcome [16–18]. A study by Swanson and Howell [19] reported a significant correlation between reading comprehension and both verbal working memory and visuospatial working memory measures. Goff and colleague [20] found a small correlation between visuospatial working memory performance and reading comprehension, but the contribution of verbal working memory was not significant. Children with reading disabilities, regardless of their verbal intelligence or concurrent arithmetic difficulties, showed lower levels of memory performance in comparison to skilled readers. This group showed pervasive memory deficit in the executive system that plays a primary role in literacy growth, when compared with the achievement level of skilled readers [21].

Interference appears to affect the close relationship between working memory and high levels of cognitive efficiency [22]. Several studies have reviewed academic development in children, and the findings indicated that children with poor reading comprehension are the prototypical example of a learning disability strictly associated with a working memory deficit with the failure to control intrusion by irrelevant information [23, 24]. The presence of processing distractors enforces stronger encoding into working memory than merely attending to them [25]. Measures of controlled attention were found to show a substantial correlation with measures of (visual) working memory when no irrelevant stimuli were presented and no contents needed to be removed from the working memory [26].

From a review of current assessment procedures, we found that the assessments were not always appropriate for the measurement of cognitive ability as regards provision of clues for enhanced occupational therapy treatment. This was because nearly all tools used involved neurocognitive assessments to identify and predict cognitive deficit. There have also been limitations in using the assessments in an occupational therapy setting because most of the working memory assessments have been developed by workers in psychological and neurological fields. Therefore, occupational therapists have the competencies necessary to use these assessments to measure working memory performance of children with the aim of informing an occupational intervention plan.

The format of the visuospatial working memory assessment constructed in this research consists of two parts, a computerized and a tabletop test. There are benefits to both forms of assessment. Computer assessments provide utilizable data effectively and quickly with a consistent presentation of stimuli to all participants. It can therefore limit experimental errors and can be a convenient method for nonexpert assessors, with a user-friendly interface. However, in the case of the tabletop test, there are some advantages in that a real object task enables children to perform the tasks on their own with objects used in the assessment. This effect is stronger as there is an aspect of realism in the eyes of children. The tabletop test was designed in the form of a board game, which is a novel approach at the present time and has been found to encourage the children to participate in the test. This pragmatic approach facilitates the use of the test by occupational therapists in clinical assessment.

Undoubtedly, working memory is generally regarded as having finite capacity [3, 27]. According to Cowan [7], young adults have approximately four chunks of working memory, while children and older adults have fewer blocks. Thus, working memory test measures are crucial in the determination of cognitive functioning and fundamental skills necessary for academic learning [28]. However, at present, there are a variety of tasks that researchers usually prefer to use to determine working memory performance. In particular, the *N*-back and running memory tasks are frequently used to test visuospatial working memory, one component of working memory. Researchers currently prefer the *N*-back task in studies into working memory because it taps into processes involving manipulation as well as maintenance of information in working memory (e.g., [29–31]). Additionally, the mechanism behind running memory span is also of recent interest because this task has led to competing inferences about working memory [32–34].

It is well-known that visuospatial working memory is the main part of working memory essential to daily occupational performance of children. It enables them to encode or update the spatial position of objects, as well as to retain and manipulate the information to perform such daily activities as reading, writing, and mathematics [1, 5, 6]. Therefore, the research team was interested in developing a visuospatial working memory assessment for children aged 7-12 years, a critical period in the life of a child as regards the development of these skills. This study is aimed at outlining the development and examining the psychometric properties of the VWMA in terms of content validity, internal consistency, test-retest reliability, and construct validity.

## 2. Materials and Methods

The methodology for the study comprised three phases. Phase I presents the theoretical conceptualizations of assessment development. Phase II describes expert evaluation methods to examine the content validity of the developed VWMA. Phase III describes the examination of psychometric properties of assessment tools in terms of internal consistency, reliability, and test-retest reliability. The details of the methodology in each study phase are as follows.

### 2.1. Phase I: Theoretical Conceptualization of Assessment Development

The VWMA was developed based on Baddeley's Working Memory Model [1, 35] as part of visuospatial sketchpad. The concept of *N*-back and running memory tasks was applied in visuospatial form to design the items of the visuospatial working memory test. Details concerning two aspects of the VWMA, specifically the *N*-back and running memory tasks, will be described later. The assessment included the aspect of interference in three experiments in the test: noninterference, auditory interference, and visual interference. The VWMA was divided into two parts: the computerized test and the tabletop test. The computerized test included automated administration, scoring, and an interpretation process. The participants were presented with stimuli on a computer screen and were instructed to respond to the tasks. The tabletop test was presented in a board game format whereby children were tested by following the instructions of an examiner. In this test, evaluation of visual processing was carried out using realistic objects. Both the computerized and tabletop aspects of the VWMA included 18 items.

During the development of any test, in this case the psychometric assessment, test-retest reliability is crucial in establishing the validity in construct of the assessment. The most straightforward method for determining the reliability of test scores is to administer the identical test twice to the same group of heterogeneous and representative subjects and compare the resulting data. If the test is perfectly reliable, each person's second score will be completely predictable from his or her first score [36]. In addition, Devellis [37] suggested that the reliability of a set of items administered on two occasions can be estimated by the correlation between the scores from the two occasions.

### 2.2. Phase II: Expert Evaluation Method

To examine the content validity of the developed VWMA, a team of experts judged the methods used. The first draft of the developed assessment tool was sent to five experts across the areas of neuropsychology, special education, and occupational therapy. The experts included a pediatrician, a psychologist, a teacher, a special education teacher, and an occupational therapist, who participated in this study for the construct of visuospatial working memory performance. The five experts were asked to rate each item of the assessment tools using the following scale: agree (1), disagree (-1), or not sure (0). The rating of all items was calculated for its Item Objective Congruence index (IOC). The values of IOC between 0.05 and 1 were accepted. After that, all items of the assessment were revised and improved according to suggestions from the experts. The researchers then modified the assessments in accordance with any suggestions by the experts. Finally, the efficacy of assessment was appraised in a pilot study of a sample group of 10 children. The results from this pilot study led to the approval of the assessments.

### 2.3. Phase III: Examination of the Psychometric Properties of the Assessment Tool (Internal Consistency, Reliability, Test-Retest Reliability, and Construct Validity)

The aim of the research methodology in this phase was to achieve internal consistency, reliability, test-retest reliability, and construct validity. The details of the procedure are as follows:

#### 2.3.1. Participants

The participants included sixty normal children and thirty children with ADHD aged between 7 and 12 years old, all participants being selected using purposive sampling. The researcher contacted the director and the teachers of the school to receive the children's information. After that, the parents of the children were contacted to seek their consent for participation in this study. The research study was approved by the ethics committee. In this study, all participants had an intelligence quotient level within the average range of the TONI-4 measurement [38].

#### 2.3.2. Materials and Procedure

To examine the internal consistency and test-retest reliability in Phase III, the material in both computerized and tabletop test aspects of the VWMA was included. The format and the procedures involved in the computerized and tabletop tests are described below.

#### 2.3.3. Computerized Test

The computerized test comprised an *N*-back and running memory test.

**Figure 1:**
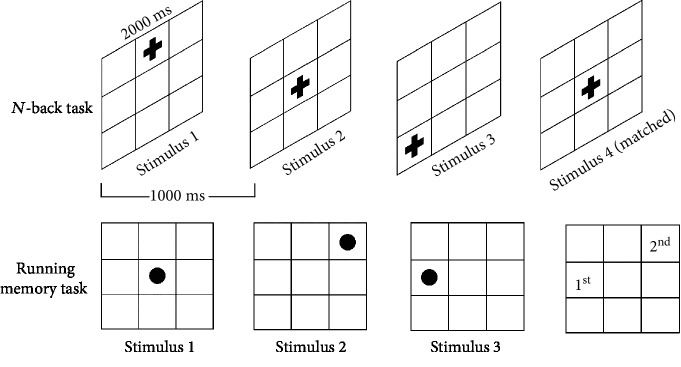
Example of the *N*-back and running memory task in the computerized test.

*(1) *N*-back Task*. In the *N*-back task, as shown in Figure 1, the participant was presented with continuous stimuli in a 9 × 9 grid on the computer screen at a viewing distance of approximately 50 cm. Each stimulus was presented for 2000 milliseconds in a specific sequential order (30–50 stimuli). The participant was requested to click on the red button if the stimulus shown was a red cross and appeared in the same position as *n* (1, 2, 3) stimulus back. In the *N*-back with auditory interference task, a dinging sound was heard as each stimulus appeared. A yellow cross was the visual interference in the *N*-back with visual interference task. The assessment included three levels of tests: 1-back, 2-back, and 3-back tasks.

*(2) Running Memory Task*. In the running memory task, as shown in Figure 1, the participant was presented with stimuli in the 9 × 9 grid on the computer screen at about 50 cm of a viewing distance. The grid was shown with a red dot appearing in any space for 2000 milliseconds (4-8 times) and then it disappeared. After the red dot vanished, the computer screen showed a blank grid. Then, the participant was requested to recall and click on the last 1, last 2, or last 3 positions where the red dot appeared. In the running memory with auditory interference task a ding sound could be heard as each stimulus appeared. A yellow dot was the visual interference in the running memory with visual interference task. The assessment included three levels of tests: 1 last running memory, 2 last running memory, and 3 last running memory tasks.

#### 2.3.4. Tabletop Test

**Figure 2:**
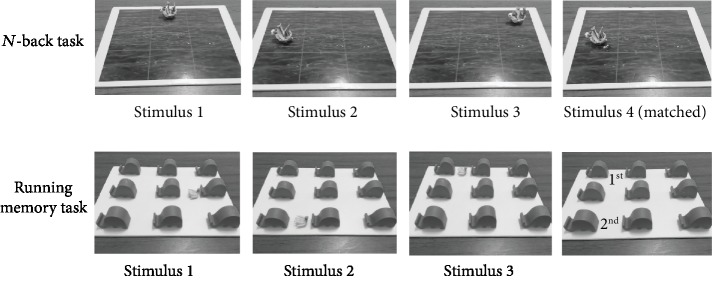
Example of the *N*-back and running memory task in the tabletop test.

*(1) *N*-back Task*. In the Pirate Ship game, as shown in Figure 2, the participant was presented with the 9 × 12 inch sea view board which was divided into nine grids. In this game, the examiner placed and removed the ship in sequential order left in position for 2000 milliseconds (12–15 times). During the game, the participant was instructed to leave a bomb by the pirate ship on any space of the sea board immediately if the ship had returned to the same *n* (1, 2, 3) position. In the *N*-back with auditory interference task, a ding sound could be heard as each ship moved. Another ship was the visual interference in the *N*-back with visual interference task. The assessment included three levels of tests: 1-back, 2-back, and 3-back tasks.

*(2) Running Memory Task*. In the Feeding Elephant game, as shown in Figure 2, the participant was presented with nine elephant models, each 3 inches in size. The examiner placed a model banana in front of an elephant model *n* times (4-7) for 2000 milliseconds mimicking the feeding of each elephant. When the examiner was finished, the participant was instructed to recall and place a banana on the last 1, last 2, or last 3 positions in sequence. In the running memory with auditory interference task, a ding was sounded as each yellow banana was moved. A green banana was used as the visual interference in the running memory with visual interference task. The assessment included three levels of tests: 1 last running memory, 2 last running memory, and 3 last running memory tasks.

#### 2.3.5. Procedure

The methodology of Phase III was to examine the psychometric properties of assessment in terms of internal consistency, test-retest reliability, and construct validity. The computerized and tabletop tests of VWMA were administered to 60 normal children to compile data to examine the internal consistency and test-retest reliability. After the data collection, Cronbach's alpha was analyzed to establish the internal consistency reliability of the assessment. Two weeks later, the same group of children was reevaluated using the same assessment to examine the test-retest reliability. The intraclass correlation coefficient (ICC) was used to examine consistency of the test-retest reliability of the assessment.

For construct validity, Cronbach's alpha coefficient equals to zero in the null hypothesis was used to determine a sample size of participants in this study [39]. In addition, the computerized and tabletop tests of VWMA were also administered to the 30 children with ADHD, the investigation group. The data from 30 age-matched children from the control group of 60 was compared with the 30 children with ADHD, the investigation group. In the first instance, the data from 30 normal children and 30 children with ADHD was used to examine the distribution of the sample data. If the data was normally distributed, an independent sample *t*-test was used to assess the statistical significance of the testing of the hypothesis. The Wilcoxon signed-rank test was used to compare two populations when the data were not normally distributed.

## 3. Results

This study is aimed at reporting the development of a new VWMA and the psychometric properties in terms of content validity, internal consistency, test-retest reliability, and construct validity.

### 3.1. Content Validity

The item-objective congruence (IOC) was used to examine the content validity of the VWMA. Thirty-six items of the assessment were examined to evaluate content validity with indices of IOC for multidimensional items. The results indicated that the VWMA had excellent content validity in both types of test. The IOC among five raters was 1.0 in overall items of the assessment, with highly rated items indicating the construct of visuospatial working memory performance. However, there were some useful suggestions offered by the experts as regards completion time, interference figure, objects, and number of subtests of each item. Consequently, the assessment was revised following the suggestions by the experts, which resulted in a higher content validity.

The modifications of assessment based on the experts' suggestions were in both the computerized and tabletop tests. In the computerized test, the visual interference figure of the *N*-back task was changed from a green cross to a yellow cross. The number of stimuli in each subtest was increased based on the difficulty level of the *N*-back task. The completion time was changed to correspond to the number of stimuli in each task. In the tabletop test, the number of items in the *N*-back task was reduced to two sets in each task. Therefore, the completion time was changed to correspond to the number of the sets of the tasks. Also, in the running memory task, the number of items was reduced to five items. The completion time was thus changed to be consistent with the number of sets of the tasks. The team of experts also suggested that the baskets for keeping bananas in front of the elephants could interfere with the visuospatial working memory performance of the children; therefore, the baskets were removed, and only bananas were placed on the board.

### 3.2. Internal Consistency

Cronbach's alpha coefficient was used to examine the internal consistency of the assessment, the results are shown in Table 1. The results show that the computerized test was found to have good reliability (18 items; *α* = .88). The Cronbach alpha scores for 9 *N*-back and 9 running memory items were .91 and .75, respectively.

Similarly, the tabletop test was found to have good reliability (18 items; *α* = .81). Cronbach's alpha scores for 9 *N*-back and 9 running memory items were .80 and .74, respectively.

### 3.3. Test-Retest Reliability

The test-retest reliability of the VWMA was evaluated through the comparison of the score correlation of the same test done by the same participant on two different occasions. Pearson's correlation coefficient was used to determine the reliability of the assessment.

The intraclass correlation coefficient was used to examine test-retest reliability of the assessment as shown in Table 2. The results showed that the computerized test was found to have excellent reliability across all aspects. In addition, the results revealed that the tabletop test was found to have fair to good reliability.

### 3.4. Construct Validity

The nonparametric Kolmogorov-Smirnov test was used to compare the two groups, the control and investigation groups. The results indicated that the mean total scores of the control group and the children with ADHD were normally distributed. There was a significance in overall tasks (*p* = .06), *N*-back task (*p* = .20), and running memory task (*p* = .06). An independent *t*-test was used to compare the mean scores of the children with ADHD and the control group. The outcomes are shown in Table 3.

The independent sample *t*-test was conducted to compare the visuospatial working memory performance of the control group of children and children with ADHD. The results indicate that the tasks of the computerized test revealed a significant difference between the two groups, the exception being the *N*-back task, as shown in Table 3. There was a significant difference in the *N*-back with auditory interference and *N*-back with visual interference task between the two groups as shown in Table 3.

In the tabletop test, the results showed that overall, the tasks of the tabletop test indicate a significant difference between the two groups as shown in Table 3. There was a significant difference in *N*-back, *N*-back with auditory interference, and *N*-back with visual interference task between the two groups as shown in Table 3.

## 4. Discussion

The purpose of developing the assessments was to detect the performance of children in order to encourage learning and sustain visuospatial working memory function, which will raise the awareness of both parents and teachers regarding the child's performance level. It will enhance the understanding of problems that could interrupt the child's performance in both occupational and daily activities. It will also offer appropriate treatment, early intervention, and suggestions for ongoing treatment in occupational therapy. The present study is aimed at developing and examining psychometric properties as regards content validity, internal consistency, test-retest reliability, and construct validity of this new visuospatial working memory assessment, which includes both computerized and tabletop formats.

Content validity is concerned with whether the content of a test elicits a range of responses that are representative of the entire domain or universe of skills, understanding, and other behaviors which a test is designed to measure. Responses to the sample of items on a well-designed test are presumably indicative of what the responses would be for the entire universe of behaviors of interest [40]. Furthermore, Devellis [37] noted that content validity is related to the definition of the construct being examined within which the contents of a scale should reflect the conceptual definition applicable to that scale. The VWMA was found to have a high level of content validity as regards construction of visuospatial working memory theory, indicating that the content validity of the developed assessment was adequate.

There are possible reasons for the excellent IOC recorded in this study. First, the assessment tool was developed based on sound theory associated with working memory and an extensive review of literature. This VWMA was developed on the basis of Baddeley's working memory model [41] including the visuospatial sketchpad. The concepts behind the tasks developed were based on the theory of the respected *N*-back [42] and running memory span tasks [43]. Second, the format of the tests, including the instruction, the scoring system, and the pictures used, was developed in a standardized manner. The items of assessment are representative of the universe of behaviors which are developed to measure visuospatial working memory performance. The results of the study revealed that the VWMA had an appropriate level of content validity. This evidence adds weight to the usefulness of the assessment, supported by peer review as it agrees with Gregory [36] who noted that content validity serves as a crucial concept when there is extensive knowledge about the variable under assessment. The achievement test contributes to the possibility of specifying the relevant universe of behavior.

The internal consistency is beneficial for the evaluation of the test homogeneity, which can be established if the items within the test can measure a single trait, meaning they are unifactorial [44]. It is also concerned with the homogeneity of the items within a scale. The scales based on classical measurement models are intended to measure a single phenomenon [37]. Cronbach's alpha coefficient was used to examine the internal consistency of the VWMA in both the computerized and tabletop tests. The results revealed good internal consistency reliability in both the computerized and tabletop tests. These results indicated that all items within the VWMA could measure the same construct of visuospatial working memory performance. The results of high internal consistency reliability support the strength of the test development based on the theory that an assessment measures the single factor among all items. Miller et al. [45] have suggested that internal consistency is a measure of how related the items (or groups of items) are to each other. Another way to think about it is whether knowledge of how a person answered one item on the test would give you information that would help you correctly predict how he or she answered another item on the test.

The results of this study indicated that the VWMA illustrated an adequate Cronbach's alpha coefficient total score, suggesting that the assessment was developed to measure the single factor among all items. The homogeneity characteristic of the assessment describes a relativity between all items of the assessment which are measured to predict the same factor. In addition, the assessment appeared statistically to have the same construct in all of its items, indicating its quality as a tool to measure visuospatial working memory performance of children.

To examine the test-retest reliability of the assessment, the VWMA was used to examine the performance of sixty normal children twice in two weeks. An analysis of intraclass correlation coefficient (ICC) was used to determine test-retest reliability of the assessment. The results showed that the test-retest reliability of the assessment were within statistically significant limits. The reported ICC values for VWMA were excellent for the computerized test. The tabletop test was found to have a fair to good reliability. The variable that may reflect changes in test-retest reliability of the tabletop test included children's cumulative experience as children performed better as regards visuospatial working memory performance during the second examination. This is because children were likely to have gained perspective about the administration and instructions of the tabletop test, which they could then adapt and apply to the real object test. Aiken and Groth-Marnat [40] concluded that the test-retest procedure takes into account errors of measurement resulting from differences in conditions (environmental and personal) associated with the two occasions on which the test is administered.

Nevertheless, the results indicated that the assessment had stable reliability in measuring visuospatial working memory performance over time, which can be supported by several pieces of evidence. First, there was a consistency in the examination process in both the computerized and tabletop tests, in particular in the computerized test, which could enhance the administration and interpretation of the online format. The computerized test enables examiners to reduce testing time and increase test accuracy to enable faster scoring, reporting, and access to results. Also, in the case of the tabletop test, the examiners were more precise when they had received training, thereby providing accurate administration of the same test on two separate occasions. Furthermore, the content of the assessment includes a cognitive ability test involving executive function where no differences can be observed over a short period of time for any children that are perhaps not mature enough to perform complex tasks until adolescence or early adulthood (e.g., [46–48]). Therefore, the performance of each child did not differ when measured on two separate occasions. The results revealed a high level of test-retest reliability which supports the appropriate nature of the assessment as a tool to evaluate visuospatial working memory over time.

A known-group method was used to examine the construct validity of the visuospatial working memory assessment, which compared performances between children with ADHD and normal children. The results from the tasks in the computerized test showed a significant difference between the control group and children with ADHD. However, the remarkable finding was that the tasks in the computerized test showed a significant difference between the control group and the ADHD group except from the *N*-back task. It was clearly seen that children with ADHD showed the same visuospatial working memory ability as the control group. The difference regarding the *N*-back task in the computerized test was at the first level of the *N*-back task. This is a very straightforward assessment so the children could easily complete the task.

The results of the tabletop test revealed a significant difference between the control group and the children in the ADHD group overall. Clearly, the noninterference task of the *N*-back in the computerized test was not strong enough to affect the visuospatial working memory performance of children with ADHD as both groups showed the same level of performance in this task. This evidence could suggest an effect of interference that children with ADHD did not perform a visuospatial working memory performance differently on the noninterference condition of *N*-back tasks compared to normal children. However, the result revealed that children with ADHD showed lower levels of performance in the interference of visuospatial working memory tasks when compared with the control group. The children in the ADHD group had lower efficiency levels especially in the interference tasks, showed more error responses, and were easily distracted during tasks. These results supported the previous literature evidence of interference control in ADHD. Barkley [49] stated that the main deficit in ADHD was behavioral disinhibition, which divided cognitive inhibition into three interrelated procedures: first, inhibiting an originally prepotent response to an event; second, trying to prevent a pattern of continuous response or response pattern; and third, controlling of interference. Children with ADHD have usually been found to be readily distracted by irrelevant data [50], the key ADHD deficit being the inability to use inhibitory processes when necessary [49]. The results of our study showed a difference in the visuospatial working memory performance between a control group of children not diagnosed with ADHD and children with ADHD especially in conditions in which there was interference.

## 5. Conclusions

In conclusion, the results show that the VWMA developed as a result of this study had good content validity, internal consistency, test-retest reliability, and construct validity. The VWMA showed adequate preliminary psychometric properties to enable the assessment of visuospatial working memory performance in children. An important point is that the visuospatial working memory plays a crucial role in the occupational performance of children, including aspects associated with daily living and academic learning. Consequently, the VWMA will be of benefit for occupational therapists in clinical use to identify visuospatial working memory problems that could affect the occupational performance of children both at the time and in the future.

## Figures and Tables

**Table 1:** Internal consistency of assessment based on statistical analysis using Cronbach's alpha coefficient.

Assessment	Task	Cronbach's alpha coefficient (*α*)	Total Cronbach's alpha coefficient (*α*)
Computerized test	*N*-back	.91	.88
	Running memory	.75	

Tabletop test	*N*-back	.80	.81
	Running memory	.74	

**Table 2:** Test-retest reliability of VWMA based on statistical analysis using Cronbach's alpha coefficient.

Format	Task		Intraclass correlation^b^	95% confidence interval		*F* test with true value 0	
				Lower bound	Upper bound	Value	*p*
Computerized test	*N*-back	Single measures	.80^a^	.68	.87	8.84	.00
		Average measure	.89^c^	.81	.93	8.84	.00
	*N*-back with auditory interference	Single measures	.79^a^	.68	.87	8.72	.00
		Average measure	.89^c^	.81	.93	8.72	.00
	*N*-back with visual interference	Single measures	.78^a^	.66	.86	8.09	.00
		Average measure	.88^c^	.79	.93	8.09	.00
	Running memory	Single measures	.98^a^	.97	.99	117.03	.00
		Average measure	.99^c^	.99	.99	117.03	.00
	Running memory with auditory interference	Single measures	.98^a^	.96	.99	83.41	.00
		Average measure	.99^c^	.98	.99	83.41	.00
	Running memory with visual interference	Single measures	.79^a^	.68	.87	8.70	.00
		Average measure	.89^c^	.81	.93	8.70	.00

Tabletop test	*N*-back	Single measures	.44^a^	.21	.62	2.56	.00
		Average measure	.61^c^	.35	.77	2.56	.00
	*N*-back with auditory interference	Single measures	.43^a^	.20	.62	2.51	.00
		Average measure	.60^c^	.33	.76	2.51	.00
	*N*-back with visual interference	Single measures	.35^a^	.10	.55	2.06	.00
		Average measure	.51^c^	.19	.71	2.06	.00
	Running memory	Single measures	.35^a^	.11	.56	2.09	.00
		Average measure	.52^c^	.19	.71	2.09	.00
	Running memory with auditory interference	Single measures	.42^a^	.19	.61	2.46	.00
		Average measure	.59^c^	.32	.76	2.46	.00
	Running memory with visual interference	Single measures	.46^a^	.23	.64	2.70	.00
		Average measure	.63^c^	.38	.78	2.70	.00

**Table 3:** Analysis of the known-group method of the computerized test analyzed statistically using the independent sample *t*-test (*N* = 60).

Format	Task		Independent *t*-test for equality of means						
			*t*	Df	*p*	Mean difference	Std. error difference	95% confidence interval of the difference	
								Lower	Upper
Computerized test	*N*-back	*N*-back	1.61	59	.11	.74	.46	-.18	1.66
		*N*-back with auditory interference	2.13	59	.04	.96	.45	.06	1.86
		*N*-back with visual interference	2.48	59	.02	1.23	.50	.24	2.22
	Running memory	Running memory	5.34	59	<.001	1.57	.30	.98	2.16
		Running memory with auditory interference	6.23	59	<.001	1.69	.27	1.15	2.23
		Running memory with visual interference	5.86	59	<.001	1.60	.27	1.05	2.14

Tabletop test	*N*-back	*N*-back	3.52	59	<.001	1.14	.32	.49	1.78
		*N*-back with auditory interference	5.34	59	<.001	2.01	.38	1.26	2.76
		*N*-back with visual interference	3.05	59	<.001	1.07	.35	.37	1.78
	Running memory	Running memory	7.17	59	<.001	2.01	.28	1.45	2.57
		Running memory with auditory interference	8.40	59	<.001	2.27	.27	1.73	2.81
		Running memory with visual interference	8.03	59	<.001	2.19	.27	1.64	2.73

Note: ^∗^*p* < 0.05.

## Data Availability

The data that support the findings of this study are available from the corresponding author upon reasonable request.
